# Serum inhibin B/follicle-stimulating hormone ratio as a predictor of testicular pathology in azoospermic men undergoing testicular sperm extraction: a retrospective study

**DOI:** 10.7717/peerj.21822

**Published:** 2026-10-29

**Authors:** Mehmet Sezai Ogras, Kadir Yildirim, Mumin Emiroglu

**Affiliations:** 1 Department of Urology, Elazığ Fethi Sekin City Hospital Health Application and Research Center, University of Health Sciences, Elazığ, Turkey; 2 Department of Pathology, Elazığ Fethi Sekin City Hospital Health Application and Research Center, University of Health Sciences, Elazığ, Turkey

**Keywords:** Non-obstructive azoospermia, INHB/FSH ratio, Micro-TESE, Biomarker, Histopathology

## Abstract

**Background:**

Non-obstructive azoospermia (NOA) represents a severe form of male infertility characterized by impaired spermatogenesis and heterogeneous histopathological findings. Identifying candidate non-invasive biomarkers associated with underlying testicular histopathology before surgical sperm retrieval may provide additional information during preoperative evaluation and patient counseling.

**Introduction:**

This study aimed to investigate the association of serum inhibin B (INHB), follicle-stimulating hormone (FSH), and their ratio (INHB/FSH) with histopathological groups in men undergoing microdissection testicular sperm extraction (micro-TESE).

**Materials and Methods:**

A total of 73 men with NOA who underwent micro-TESE were retrospectively analyzed. Patients with obstructive azoospermia, endocrine disorders, or incomplete data were excluded. Serum FSH, luteinizing hormone (LH), testosterone, and INHB levels were measured within 2 months before surgery. Based on histopathological evaluation, patients were classified into five histopathological groups: Sertoli cell-only syndrome (SCOS), early maturation arrest (EMA), late maturation arrest (LMA), hypospermatogenesis (HSG), and normospermatogenesis (NS). The association between hormonal parameters and histopathological groups was evaluated using receiver operating characteristic (ROC) analysis and binary logistic regression.

**Results:**

The INHB/FSH ratio differed significantly among the histopathological groups (*p* < 0.001). Receiver operating characteristic analysis demonstrated moderate-to-high discriminative performance, with area under the curve values ranging from 0.77 to 0.91. In this exploratory cohort, provisional thresholds were observed across the histopathological groups, with lower values generally associated with SCOS, EMA, and LMA and higher values associated with HSG and NS. These findings should be considered hypothesis-generating and require internal and external validation before clinical application. Binary logistic regression demonstrated that higher INHB/FSH ratios were significantly associated with favorable sperm retrieval outcomes, with an odds ratio of 2.36 per 10-unit increase.

**Conclusions:**

The serum INHB/FSH ratio was associated with histopathological groups and sperm retrieval outcomes in men with NOA undergoing micro-TESE. These findings suggest that the INHB/FSH ratio may warrant further investigation as a candidate non-invasive biomarker associated with histopathological groups in men with NOA.

## Introduction

Non-obstructive azoospermia (NOA) affects approximately 5–15% of infertile men and is considered one of the most severe forms of male infertility (Hamoda et al., 2025). The etiological basis of NOA is multifactorial, and the majority of cases are regarded as idiopathic. Numerous contributing factors have been identified, including chromosomal abnormalities, genetic defects, infections, testicular trauma, varicocele, and endocrine dysfunctions. The fundamental pathophysiological mechanism involves impaired spermatogenesis at the testicular level (Wu et al., 2021). Microdissection testicular sperm extraction (Micro-TESE) is widely regarded as the preferred surgical technique for sperm retrieval in men with non-obstructive azoospermia; however, its designation as an absolute gold standard should be interpreted with caution. According to the SIAMS 2022 position statement, the choice between micro-TESE and conventional TESE may depend on center expertise, surgeon experience, and available resources (Ferlin et al., 2022). The 2021 American Urological Association (AUA)/American Society for Reproductive Medicine (ASRM) guideline recommends micro-TESE as the preferred sperm retrieval technique for men with NOA (Schlegel et al., 2021). The procedure enables sperm retrieval in approximately 40–60% of patients while minimizing unnecessary tissue excision (Kong et al., 2021). Given the substantial inter-individual variability in sperm retrieval outcomes, identifying candidate non-invasive biomarkers associated with underlying spermatogenic activity may provide additional information during preoperative assessment and patient counseling (Pozzi et al., 2024). Extensive research has focused on the relationship between hormonal biomarkers and sperm retrieval outcomes. Owing to their essential roles in spermatogenesis and Sertoli cell maturation, follicle-stimulating hormone (FSH) and serum inhibin B (INHB) are widely recognized as important endocrine indicators (Ni, Hao & Yang, 2019). INHB, secreted by Sertoli cells, regulates pituitary FSH release through a negative feedback mechanism. Disruption of seminiferous epithelial integrity is associated with reduced INHB concentrations and elevated FSH levels (Achermann & Esteves, 2023). This reciprocal physiological relationship suggests that the INHB/FSH ratio may reflect spermatogenic capacity more comprehensively than either parameter alone.

The primary aim of this study was to evaluate the association of serum INHB, FSH, and the INHB/FSH ratio with histopathological groups in patients with NOA undergoing micro-TESE.

## Materials and Methods

### Study design and ethical approval

This retrospective, single-center study was conducted at Elazığ Fethi Sekin City Hospital (Türkiye), between January 2023 and September 2025. The study protocol was approved by the Non-Interventional Ethics Committee of the same institution (Approval No: 2025/12-24). The study was performed in accordance with the ethical principles of the Declaration of Helsinki. Written informed consent was obtained from all patients before surgery, and the consent form explicitly stated that their clinical data could be used for scientific and clinical research purposes, in accordance with applicable ethical and institutional regulations. The study protocol was registered at ClinicalTrials.gov (Identifier: NCT07415928). This study was designed and reported in accordance with the Strengthening the Reporting of Observational Studies in Epidemiology (STROBE) guidelines. A detailed flow diagram illustrating patient inclusion, classification based on histopathology, and data analysis steps is presented in Fig. 1.

**Figure 1:**
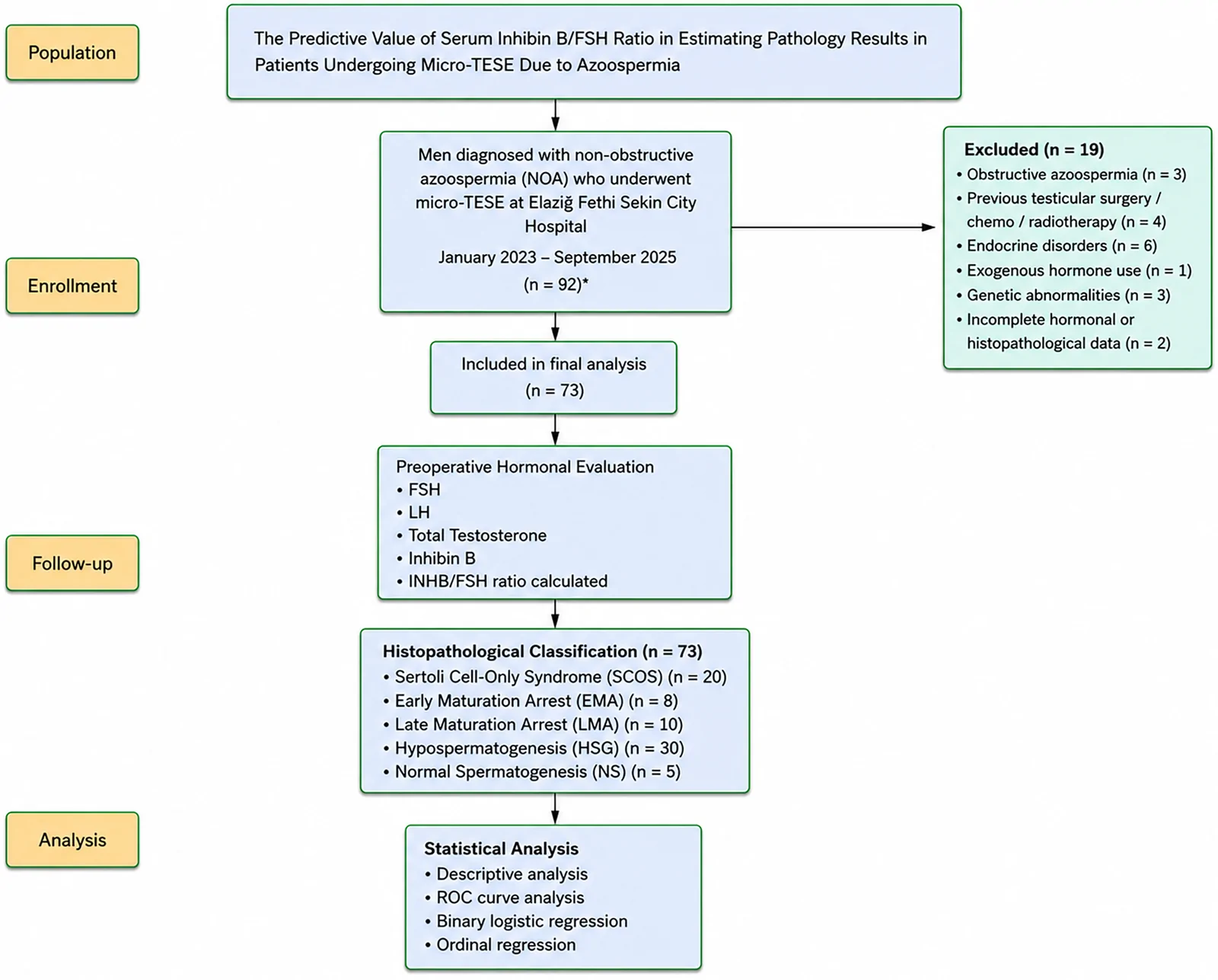
Flow diagram of patient selection, histopathological classification, and statistical analysis.

### Patient selection

This study included patients diagnosed with NOA who underwent micro-TESE. Demographic, hormonal, and histopathological data were collected retrospectively from the hospital’s electronic medical records.

#### Inclusion criteria

- Diagnosis of NOA confirmed by at least two semen analyses showing complete absence of spermatozoa after centrifugation.
- Between 20 and 50 years of age,
- Underwent micro-TESE,
- Availability of complete preoperative hormonal data (FSH, LH, total testosterone, INHB) measured within 2 months before surgery.
- Availability of histopathological evaluation results after micro-TESE.

#### Exclusion criteria

- Obstructive azoospermia (OA) or ejaculatory duct obstruction diagnosed *via* clinical, biochemical, or imaging findings.
- History of previous testicular surgery, chemotherapy, radiotherapy, or testicular trauma.
- Presence of systemic endocrine disorders (*e.g*., hypogonadotropic hypogonadism, thyroid dysfunction, pituitary adenoma).
- Use of exogenous testosterone, anabolic steroids, or hormonal therapy within the past 6 months.
- Incomplete medical records, missing hormonal data, or unavailable histopathology results.
- Coexisting genetic abnormalities confirmed through karyotype or molecular analysis.

### Protocol and outcome measures

#### Surgical technique

The procedure is based on the use of an operating microscope and microsurgical techniques to identify and retrieve seminiferous tubules with a higher likelihood of containing spermatozoa. After spinal anesthesia with intravenous sedation was established. A midline scrotal incision was made and the testis was delivered through the incision. The tunica vaginalis was opened, and the tunica albuginea was incised under the operating microscope. The seminiferous tubules were examined macroscopically. Tubules that appeared larger and more opaque—features suggestive of a higher probability of containing spermatozoa were identified. Small tissue samples were excised using microsurgical scissors with minimal trauma to the surrounding tissue. The excised specimens were immediately transferred to the embryology laboratory, where the presence or absence of spermatozoa was confirmed by direct microscopic examination. At the conclusion of the procedure, the tunica albuginea and scrotal layers were closed with sutures. Sperm retrieval success was defined as the presence of at least one viable spermatozoon identified in the retrieved testicular tissue sample during micro-TESE. Patients were discharged after achieving an Aldrete score ≥9 according to the institutional post-anesthesia recovery protocol.

#### Histopathological examination

The obtained testicular biopsy specimens were fixed in 10% neutral buffered formalin for at least 6 h. Following fixation, tissue processing was performed according to the standard histopathological protocol. During dehydration, the specimens were passed through a graded series of ethanol (70%, 96%, and 100%) for a total of approximately 2–3 h. Subsequently, in the clearing phase, the tissues were treated twice with xylene for a total duration of 40–60 min. For paraffin infiltration, the samples were processed in two stages in molten paraffin baths, each lasting approximately 45–60 min, and then embedded into paraffin blocks. Sections were cut at a thickness of 3 µm using a rotary microtome, mounted on glass slides *via* a water bath, and dried at 37 °C for at least 24 h. Hematoxylin and eosin (H&E) staining was employed for routine histological evaluation. Prepared slides were examined under a Leica DM2000 LED light microscope by a single experienced pathologist in a blinded manner. Histopathological diagnoses were established according to the following morphological criteria: Sertoli cell-only syndrome (SCOS) was defined by the presence of only Sertoli cells in all seminiferous tubules with a complete absence of germ cells. Early maturation arrest (EMA) was characterized by cessation of spermatogenesis at the spermatogonium or primary spermatocyte stage, without the presence of more advanced germ cells. Late maturation arrest (LMA) was defined by germ cell development reaching the spermatid stage, but without mature spermatozoa in the tubules. Hypospermatogenesis (HSG) was diagnosed when all stages of spermatogenesis were present but with a marked reduction in germ cell numbers and thinning of the germinal epithelium in the seminiferous tubules. Normospermatogenesis (NS) was defined as preserved spermatogenesis with complete maturation and the presence of spermatozoa in the seminiferous tubules.

#### Histopathological classification

Based on the results of testicular histopathology, patients were categorized into five groups:

(1) Early Maturation Arrest (EMA)
(2) Late Maturation Arrest (LMA)
(3) Hypospermatogenesis (HSG)
(4) Normospermatogenesis (NS)
(5) Sertoli Cell-Only Syndrome (SCOS)

All evaluations were performed by the same pathologist, blinded to hormonal results, to minimize observer bias.

#### Hormonal measurements

There was variability in the timing of preoperative hormonal measurements, which were obtained within a defined preoperative window (2 months before surgery). Serum hormone levels, including FSH, LH, testosterone, and INHB, were measured using the Beckman Coulter DXI 800 Immunoassay Analyzer based on the chemiluminescence method. In patients with serum inhibin B levels below the assay’s lower limit of detection (4.6 pg/mL), the minimum detectable value was assigned for statistical analysis. This affected nine patients, and the value represents the assay’s lower quantification limit rather than true biological zero.

### Sample size

All eligible patients treated during the study period who met the inclusion criteria and had complete data were included (*n* = 73). No formal sample size calculation was performed, as the study was designed as a retrospective exploratory analysis including all available cases. The study was designed for both descriptive and exploratory purposes. The diagnostic performance was assessed by presenting the obtained area under curve (AUC) values along with their 95% confidence intervals to reflect the accuracy of the predictions. While the current sample size is adequate for preliminary evaluation, further prospective studies with larger sample sizes are needed to validate the findings.

### Statistical analysis

All statistical analyses were performed using SPSS version 25.0 (IBM Corp., Armonk, NY, USA). Descriptive statistics were used to summarize patient characteristics and sperm retrieval outcomes across histopathological groups. Continuous variables were expressed as mean ± standard deviation (SD) or median (interquartile range, IQR), while categorical variables were presented as frequencies and percentages.

Comparisons of the INHB/FSH ratio among histopathological groups were performed using the Kruskal–Wallis test or one-way analysis of variance (ANOVA), depending on data distribution.

The discriminative performance of the INHB/FSH ratio for identifying each histopathological group was evaluated using receiver operating characteristic (ROC) curve analysis. The AUC, optimal provisional thresholds (determined by the Youden index), and the corresponding sensitivity and specificity were calculated. Because the ROC-derived provisional thresholds were generated and evaluated within the same cohort, they were considered exploratory and intended to generate hypotheses, and require internal and external validation before any potential clinical application.

To assess the association between the INHB/FSH ratio and sperm retrieval success, binary logistic regression analysis was performed by classifying patients into two groups according to sperm retrieval outcomes (low retrieval: SCOS, EMA, and LMA; high retrieval: HSG and NS). For improved clinical interpretability, odds ratios were presented per 10-unit increase in the INHB/FSH ratio.

A *p*-value of <0.05 was considered statistically significant for all analyses. Bonferroni correction was applied for the five ROC subgroup analyses (SCOS, EMA, LMA, HSG, and NS), resulting in an adjusted significance threshold of *p* < 0.01 (0.05/5). ROC findings were interpreted according to this adjusted threshold, and no false discovery rate (FDR) correction was applied.

## Results

The study included a total of 73 patients who underwent micro-TESE. The mean age of the participants was 32.37 ± 5.67 years (range: 22–47). The mean serum FSH level was 10.92 ± 10.22 mIU/mL (range: 2.10–58.22), while the mean INHB concentration was 124.56 ± 97.84 pg/mL (range: 4.6–379.7). In nine patients, INHB levels were below the lower limit of detection (4.6 pg/mL). The calculated INHB/FSH ratio showed a skewed distribution, with a mean ± standard deviation of 27.14 ± 30.18 (range: 0.12–118.38) and a median (interquartile range, IQR) of 18.6 (6.2–42.8).

### Distribution of histopathological groups and sperm retrieval success

The distribution of patients across different histopathological groups revealed substantial differences in sperm retrieval success. Among the histopathological groups, HSG demonstrated the highest sperm retrieval success, with 80% of patients (24/30) achieving successful sperm retrieval. In contrast, the SCOS group demonstrated the lowest sperm retrieval success, with only 10% (2/20) of patients achieving successful sperm retrieval. EMA demonstrated no sperm retrieval success, whereas the LMA group demonstrated a sperm retrieval success of 40% (4/10). The NS group demonstrated successful sperm retrieval in all patients (5/5). Overall, the sperm retrieval success rate for the study population was 47.9%, indicating that HSG and NS were associated with higher sperm retrieval success than SCOS and EMA (Table 1).

**Table 1:** Distribution of pathology groups and sperm retrieval rates.

Pathology group	Number of patients (*n*)	Percentage (%)	Number of successful sperm retrievals (SR)	SRR (%)
SCOS	20	27.4	2	10
HSG	30	41.1	24	80
EMA	8	11.0	0	0
LMA	10	13.7	4	40
NS	5	6.8	5	100
Total	73	100	35	47.9

**Note:**

SCOS, sertoli cell-only syndrome; HSG, hypospermatogenesis; EMA, early maturation arrest; LMA, late maturation arrest; NS, normospermatogenesis; SRR, sperm retrieval rate.

### ROC analysis by histopathological group (INHB/FSH ratio)

ROC analysis demonstrated numerically similar discriminative performance of the INHB/FSH ratio across the different histopathological groups (Table 2).

**Table 2:** ROC analysis by pathology group (INHB/FSH ratio).

Pathology group	AUC	95% CI	Cut-off (Youden ındex)	Sensitivity (%)	Specificity (%)	*p*-value
SCOS	0.97	[0.91–0.99]	<5.0	90	88	<0.001
EMA	0.80	[0.68–0.92]	<15	75	78	0.004
LMA	0.77	[0.64–0.89]	<20	70	74	0.012*
HSG	0.84	[0.73–0.94]	>10	79	81	<0.001
NS	0.91	[0.82–0.99]	>45	88	85	<0.001

**Notes:**

* The LMA subgroup did not remain statistically significant after Bonferroni correction (adjusted significance threshold: *p* < 0.01).

SCOS, sertoli cell-only syndrome; EMA, early maturation arrest; LMA, late maturation arrest; HSG, hypospermatogenesis; NS, normospermatogenesis; AUC, area under the receiver operating characteristic curve; CI, confidence ınterval.

The SCOS group demonstrated an AUC of 0.89 (95% CI [0.77–0.91]). In this exploratory cohort, a provisional threshold of <5.0 was observed for SCOS. This finding should be considered exploratory and requires internal and external validation. Within the present cohort, this exploratory provisional threshold demonstrated a sensitivity of 85% and a specificity of 82% (*p* < 0.001).

The EMA group demonstrated an AUC of 0.80. In this exploratory cohort, a provisional threshold of <15 was observed for EMA. This finding should be considered exploratory and requires internal and external validation (*p* = 0.004).

The LMA group demonstrated an AUC of 0.77. In this exploratory cohort, a provisional threshold of <20 was observed for LMA. However, this result did not remain statistically significant after Bonferroni correction (adjusted significance threshold: *p* < 0.01).

The HSG group demonstrated an AUC of 0.84. In this exploratory cohort, a provisional threshold of >10 was observed for HSG. This finding should be considered exploratory and requires internal and external validation.

The NS group demonstrated an AUC of 0.91. In this exploratory cohort, a provisional threshold of >45 was observed for NS. This finding should be considered exploratory and requires internal and external validation before any potential clinical application. Sensitivity and specificity for this provisional threshold were 88% and 85%, respectively. These performance estimates should also be interpreted as exploratory because they were derived and evaluated within the same cohort (Table 2, Fig. 2).

**Figure 2:**
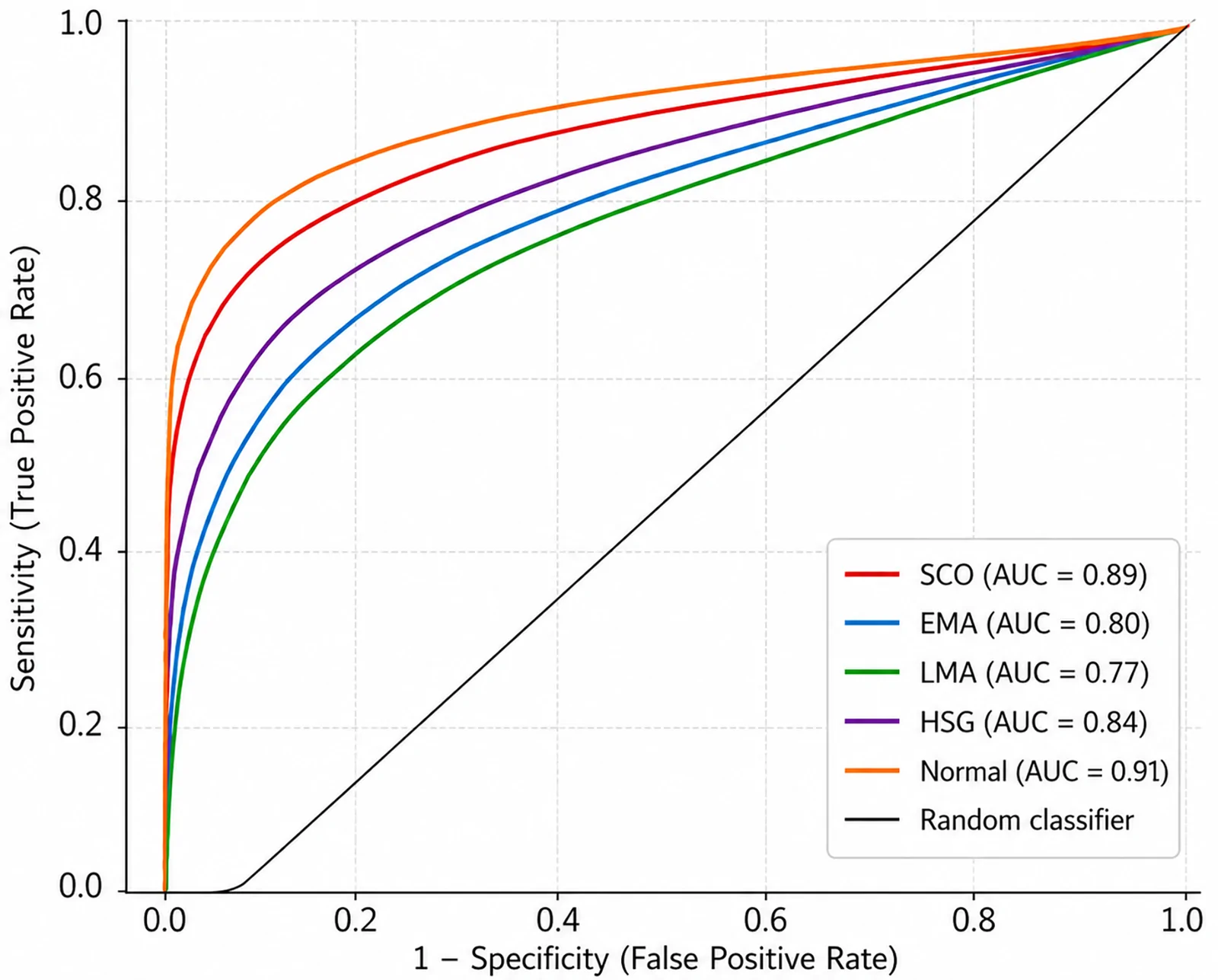
One-*vs*-rest ROC curves for INHB/FSH ratio according to histopathological groups.

### Binary logistic regression

Binary logistic regression was performed by classifying patients into two groups according to sperm retrieval outcomes:

- Low sperm retrieval success: SCOS, EMA, and LMA
- High sperm retrieval success: HSG and NS

When expressed per 10-unit increase, the odds ratio was 2.36, providing a clinically interpretable estimate of the association between the INHB/FSH ratio and sperm retrieval success. Higher INHB/FSH ratios were significantly associated with greater sperm retrieval success (Table 3). These findings demonstrate a significant association between higher INHB/FSH ratios and favorable sperm retrieval outcomes in this retrospective exploratory study.

**Table 3:** Binary logistic regression analysis.

Parameter	β (coefficient)	OR (Exp(β))	95% CI	*p*-value	Interpretation
Intercept	−1.92	–	–	0.002	Represents the baseline log-odds of sperm retrieval.
INHB/FSH Ratio	0.085	1.089	[1.045–1.135]	<0.001	Higher INHB/FSH ratios were associated with an increased probability of favorable spermatogenesis and successful sperm retrieval.

**Notes:**

β, regression coefficient; OR, odds ratio; CI, confidence interval; INHB/FSH, inhibin B-to-follicle-stimulating hormone ratio. A *p* value < 0.05 was considered statistically significant.

## Discussion

The Inhibin B/FSH ratio provides an integrated signal of Sertoli cell function and spermatogenesis. Current evidence suggests that this ratio is useful in reflecting impaired spermatogenesis. In the present study, unlike previous reports in the literature, we specifically investigated whether the INHB/FSH ratio has predictive value for testicular pathology. INHB is secreted by Sertoli cells and regulates pituitary FSH secretion *via* a negative feedback mechanism (Leslie, Mejias & Ramphul, 2025). As INHB decreases, FSH typically increases; this inverse relationship reflects the functional integrity of the seminiferous epithelium (Andersson et al., 2004; Anawalt, 2013). In different populations, a positive correlation has been reported between inhibin B levels and the INHB/FSH ratio with sperm concentration and total sperm count (Meeker, Godfrey-Bailey & Hauser, 2007; Pierik et al., 1998). The combined evaluation of inhibin B and FSH may provide additional information regarding impaired spermatogenesis compared with FSH alone, and the INHB/FSH ratio reflects this physiological relationship (Andersson et al., 2004; Anawalt, 2013). In cases of non-obstructive azoospermia (NOA), micro-TESE not only enables sperm retrieval but also provides direct access to testicular histopathology. Pathology reports provide critical information regarding the presence or absence of spermatogenesis (Ramasamy, Yagan & Schlegel, 2015; Shi et al., 2025). Current studies indicate that histopathological findings are partially associated with sperm retrieval outcomes; however, they are not sufficient alone to guide surgical decision-making. The most common histopathological patterns observed in micro-TESE biopsies are SCOS, LMA, EMA, HSG, and NS. These groups show variable associations with clinical biomarkers (FSH, inhibin B, AMH) and the likelihood of sperm retrieval. In SCOS, the probability of sperm retrieval is low (approximately 10–20%). In maturation arrest, sperm retrieval is very limited in early arrest, whereas retrieval may still be possible in late arrest. HSG demonstrates the highest sperm retrieval rates, reported up to 70–90% in the literature. In normal or focal spermatogenesis, localized sperm foci may be identified, representing a recognized feature of micro-TESE compared with conventional TESE (Caroppo & Colpi, 2021; Ferlin et al., 2022). In this study, we investigated the relationship between the serum INHB/FSH ratio and histopathological diagnosis in patients undergoing micro-TESE for NOA. We also evaluated the association of this hormonal ratio with histopathological outcomes and sperm retrieval success. Our findings suggest that the INHB/FSH ratio may represent a candidate non-invasive biomarker associated with histopathological patterns and sperm retrieval outcomes in this exploratory cohort. The distribution of pathology groups in our cohort confirmed the heterogeneity of spermatogenic activity in NOA. The highest sperm retrieval rates were observed in the HSG and NS groups (80% and 100%, respectively), whereas SCOS and EMA showed markedly lower rates (10% and 0%, respectively). These findings are consistent with previous studies reporting that preserved spermatogenic foci in HSG are associated with higher sperm retrieval success, whereas SCOS and EMA are associated with more severely impaired spermatogenesis.

ROC analysis demonstrated moderate-to-high discriminative performance of the INHB/FSH ratio across different histopathological groups. AUC values ranged from 0.77 to 0.91. In this exploratory cohort, a provisional threshold of <5 was observed for SCOS, whereas provisional threshold values of >10 and >45 were observed for HSG and NS, respectively. In contrast, the provisional threshold identified for LMA did not remain statistically significant after Bonferroni correction and should therefore be interpreted with particular caution. These thresholds should be considered exploratory and hypothesis-generating and require external validation before clinical application. A limitation of this ROC-based analysis is the partial overlap of threshold values among histopathological groups, indicating that the INHB/FSH ratio reflects a biological continuum rather than providing strict categorical discrimination. Therefore, the ratio should be interpreted as a probabilistic indicator rather than a definitive classification tool. The observed threshold values are biologically consistent with the inverse relationship between inhibin B production and pituitary FSH secretion; however, they should not be regarded as clinically established cut-off values. Binary logistic regression demonstrated that higher INHB/FSH ratios were significantly associated with successful sperm retrieval (*p* < 0.001). When expressed per 10-unit increase, the odds ratio was 2.36, providing a more clinically interpretable estimate of the association between the INHB/FSH ratio and favorable spermatogenic status. Our findings are consistent with previous studies suggesting that INHB alone or in combination with FSH may predict spermatogenic function. Comparative ROC analyses demonstrated numerically similar discriminative performance for the INHB/FSH ratio, INHB alone, and FSH alone. Although the INHB/FSH ratio yielded a numerically higher mean AUC than INHB alone and FSH alone (0.76 *vs*. 0.75 *vs*. 0.74), no formal statistical comparison between correlated ROC curves (*e.g*., DeLong test) was performed. Therefore, these numerical differences should not be interpreted as evidence of statistical superiority but rather as exploratory findings requiring internal and external validation. Studies by Vernaeve et al. (2003) similarly suggested that this ratio may provide complementary information regarding Sertoli cell function and spermatogenic capacity (Olumide et al., 2025). Our findings are consistent with these observations. From a clinical perspective, non-invasive prediction of histopathology before micro-TESE is of considerable interest. In this exploratory cohort, patients with lower INHB/FSH ratios, particularly those below the provisional threshold of <5, tended to exhibit histopathological patterns associated with lower sperm retrieval success, whereas higher ratios were generally associated with more preserved spermatogenesis. These observations may provide additional information during preoperative counseling but should not be used as definitive decision thresholds without external validation. This study suggests that the INHB/FSH ratio may represent a candidate non-invasive biomarker for evaluating histopathological outcomes in men with NOA. Previous studies have suggested that combined hormonal indices may reflect spermatogenic function (Vernaeve et al., 2003). Consistent with these observations, the INHB/FSH ratio demonstrated numerically favorable discriminative performance while reflecting underlying spermatogenic activity. The identification of provisional threshold ranges may provide additional information during preoperative assessment. However, given the retrospective single-center design, the relatively small subgroup sizes, the absence of external validation, and the overlap in threshold values, this ratio should not be regarded as an established clinical biomarker. These findings may complement conventional preoperative assessment but should not be interpreted as evidence of improved clinical outcomes or be used as the sole basis for clinical decision-making. Since reproductive outcomes such as pregnancy, live birth, or assisted reproductive technology success were not evaluated, no conclusions can be drawn regarding overall clinical outcomes. Therefore, the findings should be regarded as preliminary and exploratory until confirmed by prospective validation studies. This study has additional limitations, including relatively small sample sizes in certain subgroups such as EMA and NS, which may reduce statistical power. In particular, the very small sample sizes of the EMA (*n* = 8) and NS (*n* = 5) subgroups may have reduced the precision and stability of the ROC estimates despite the reported 95% confidence intervals. Moreover, the subgroup ROC analyses and derived threshold values should be interpreted cautiously because of class imbalance, small subgroup sizes, and the absence of internal or external validation methods such as bootstrap resampling, cross-validation, or independent cohort validation. Therefore, the proposed threshold values should be considered exploratory rather than clinically definitive. Furthermore, no formal statistical comparison between correlated ROC curves was performed; therefore, the observed numerical differences between the INHB/FSH ratio, INHB alone, and FSH alone should not be interpreted as evidence of superiority. The retrospective design further limits causal inference, and inter-assay variability in hormone measurements may affect reproducibility. Although the INHB/FSH ratio may represent a candidate non-invasive biomarker, its discriminative performance was numerically similar to that of INHB and FSH alone in this exploratory cohort. Therefore, the potential added value of this ratio beyond conventional hormonal markers remains uncertain and requires internal and external validation in larger independent cohorts. Future multicenter studies with standardized hormonal assays, formal ROC comparisons, and both internal and external validation are needed to confirm these findings.

## Conclusions

The present study demonstrated that the serum INHB/FSH ratio was associated with histopathological patterns and sperm retrieval outcomes in men with non-obstructive azoospermia undergoing micro-TESE. Although the INHB/FSH ratio showed encouraging discriminative performance in this exploratory cohort, the proposed threshold values were derived and evaluated within the same study population and therefore require both internal and external validation. Consequently, the INHB/FSH ratio should be regarded as a candidate non-invasive biomarker that warrants further investigation rather than a clinically established decision-making tool. Given the retrospective single-center design, the relatively small subgroup sizes, and the absence of external validation, these findings should be considered exploratory until confirmed in larger prospective multicenter studies. Future studies should also determine whether these associations translate into patient-centered outcomes, including sperm retrieval success in independent cohorts, pregnancy rates, live birth, and assisted reproductive technology outcomes.

## Supplemental Information

10.7717/peerj.21822/supp-1 Supplemental Information 1 Data.

10.7717/peerj.21822/supp-2 Supplemental Information 2 STROBE Checklist.

## Additional Information and Declarations

### Data Availability

The following information was supplied regarding data availability:

The data is available in the Supplemental Files.

The data is available at Zenodo: Oğraş, M. S., Kadir, Y., & Mümin, E. (2026). Serum Inhibin B/Follicle-Stimulating Hormone Ratio as a Predictor of Testicular Pathology in Azoospermic Men Undergoing Testicular Sperm Extraction: A Retrospective Study [Dataset]. Zenodo. https://doi.org/10.5281/zenodo.20131019.

## References

[ref-1] Achermann APP, Esteves SC (2023). Prevalence and clinical implications of biochemical hypogonadism in patients with nonobstructive azoospermia undergoing infertility evaluation. F&S Reports.

[ref-2] Anawalt BD (2013). Approach to male infertility and hormonal evaluation including inhibin B. Journal of Clinical Endocrinology and Metabolism.

[ref-3] Andersson AM, Petersen JH, Jørgensen N, Jensen TK, Skakkebaek NE (2004). Serum inhibin B and follicle-stimulating hormone levels as tools in the evaluation of infertile men: significance of adequate reference values from proven fertile men. Journal of Clinical Endocrinology and Metabolism.

[ref-4] Caroppo E, Colpi GM (2021). Prediction models for successful sperm retrieval in patients with non-obstructive azoospermia undergoing microdissection testicular sperm extraction: is there any room for further studies?. Journal of Clinical Medicine.

[ref-5] Ferlin A, Calogero AE, Krausz C, Lombardo F, Paoli D, Rago R, Scarica C, Simoni M, Foresta C, Rochira V, Sbardella E, Francavilla S, Corona G (2022). Management of male factor infertility: position statement from the italian society of andrology and sexual medicine (SIAMS): endorsing organization: Italian society of embryology, reproduction, and research (SIERR). Journal of Endocrinological Investigation.

[ref-6] Hamoda T, Shah R, Mostafa T, Pinggera GM, Atmoko W, Rambhatla A, Al Hashimi M, Çayan S, Colpi GM, Alipour H, Ko E, Zini A, Dimitriadis F, Rashed A, Park HJ, Saleh R, Toprak T, Ryzhkov A, Kadıoğlu A, Kandil H, Kalkanli A, El-Sakka AI, Calik G, Falcone M, Elbardisi H, Arafa M, Ho CCC, Pedrozo Martinez M, Binsaleh S, Motawi AT, Gherabi N, Tsujimura A, Taniguchi H, Kosgi R, Calogero AE, Shatylko T, Kim D, Thomas C, Tadros NN, Andreadakis S, Musa MU, Konstantinidis C, Preto M, Le TV, Khalafalla KM, Cannarella R, Bowa K, Balagobi B, Katz DJ, Nguyen Q, Tanwar R, Borges Junior E, Agarwal A (2025). Global andrology forum (GAF) clinical guidelines on the management of non-obstructive azoospermia: bridging the gap between controversy and consensus. World Journal of Men’s Health.

[ref-7] Kong X, Ye Z, Chen Y, Zhao H, Tu J, Meng T, Xiong C, Li H, Gong Y, Zheng L, Cheng B, Zhang Z, Xu P (2021). Clinical application value of inhibin B alone or in combination with other hormone indicators in subfertile men with different spermatogenesis status: a study of 324 Chinese men. Journal of Clinical Laboratory Analysis.

[ref-8] Leslie SW, Mejias SG, Ramphul K (2025). Sertoli cell–only syndrome.

[ref-9] Meeker JD, Godfrey-Bailey L, Hauser R (2007). Relationships between serum hormone levels and semen quality. Epidemiology.

[ref-10] Ni F-D, Hao S-L, Yang W-X (2019). Multiple signaling pathways in Sertoli cells: recent findings in spermatogenesis. Cell Death & Disease.

[ref-11] Olumide OB, Godwin AI, Etukudoh NS, Dutta S, Uchejeso OM, Titilayo JO, Isichei CO, Selowo ST, Sengupta P (2025). Evaluation of serum inhibin B and inhibin B/FSH ratio in the diagnosis of non-obstructive azoospermia and oligozoospermia. Hormone Molecular Biology and Clinical Investigation.

[ref-12] Pierik FH, Vreeburg JT, Stijnen T, De Jong FH, Weber RF (1998). Serum inhibin B as a marker of spermatogenesis. Journal of Clinical Endocrinology and Metabolism.

[ref-13] Pozzi E, Corsini C, Belladelli F, Bertini A, Negri F, Raffo M, Saccà A, Ventimiglia E, Boeri L, Fallara G, d’Arma A, Pagliardini L, Viganò P, Pontillo M, Lucianò R, Colecchia M, Ramasamy R, Montorsi F, Alfano M, Salonia A (2024). Role of follicle-stimulating hormone, inhibin B, and anti-Müllerian hormone in predicting sperm retrieval from men with nonobstructive azoospermia undergoing microdissection testicular sperm extraction: a systematic review and meta-analysis. European Urology Open Science.

[ref-14] Ramasamy R, Yagan N, Schlegel PN (2015). Microdissection testicular sperm extraction: techniques, outcomes, and histopathological correlations. Fertility and Sterility.

[ref-15] Schlegel PN, Sigman M, Collura B, De Jonge CJ, Eisenberg ML, Lamb DJ, Mulhall JP, Niederberger C, Sandlow JI, Sokol RZ, Spandorfer SD, Tanrikut C, Treadwell JR, Oristaglio JT, Zini A (2021). Diagnosis and treatment of infertility in men: AUA/ASRM guideline—part II. Journal of Urology.

[ref-16] Shi S, Lv Y, Sun J, Wang T, Wang M (2025). From histopathology to clinical success: Johnsen score as predictors in micro-TESE outcomes of idiopathic non-obstructive azoospermia. Pathology—Research and Practice.

[ref-17] Vernaeve V, Tournaye H, Osmanagaoglu K, Verheyen G, Van Steirteghem A, Devroey P (2003). Intracytoplasmic sperm injection with testicular spermatozoa is less successful in men with nonobstructive azoospermia than in men with obstructive azoospermia. Fertility and Sterility.

[ref-18] Wu X, Lin D, Sun F, Cheng CY, Cheng C, Sun F (2021). Male infertility in humans: an update on non-obstructive azoospermia (NOA) and obstructive azoospermia (OA). Molecular Mechanisms in Spermatogenesis.

