# Supplementary material for: Serum inhibin B/follicle-stimulating hormone ratio as a predictor of testicular pathology in azoospermic men undergoing testicular sperm extraction: a retrospective study
Source: PeerJ. 2026 Oct 29;14:e21822. doi: 10.7717/peerj.21822 (PMC13724047; doi:10.7717/peerj.21822)
Supplement: Supplementary file 1 [file peerj-14-21822-s002.odt]

STROBE Statement—checklist of items that should be included in reports of observational studies


Item No.
Recommendation
Page 
No.
Relevant text from manuscript
Title and abstract
1
(a) Indicate the study’s design with a commonly used term in the title or the abstract
2
A total of 73 men with NOA who underwent micro-TESE were retrospectively analyzed.
(b) Provide in the abstract an informative and balanced summary of what was done and what was found
2
This study evaluated the clinical performance of the INHB/FSH ratio in predicting testicular histopathology among 73 patients with non-obstructive azoospermia (NOA). The findings indicate that this ratio can differentiate between various pathological types with high accuracy (AUC 0.77–0.91), and that increasing ratios are significantly associated with less severe histopathological categories ($p < 0.001$). By establishing specific diagnostic thresholds for stages ranging from SCOS to normal spermatogenesis, the research presents a robust biomarker for use prior to micro-TESE surgery.
Introduction

Background/rationale
2
Explain the scientific background and rationale for the investigation being reported
	3
Non-obstructive azoospermia (NOA) affects approximately 5–15% of infertile men and is considered one of the most severe forms of male infertility (Hamoda et al, 2025). The etiological basis of NOA is multifactorial, and the majority of cases are regarded as idiopathic. Numerous contributing factors have been identified, including chromosomal abnormalities, genetic defects, infections, testicular trauma, varicocele, and endocrine dysfunctions. The fundamental pathophysiological mechanism involves impaired spermatogenesis at the testicular level (Wu et al, 2021).
Microsurgical testicular sperm extraction (m-TESE) is the gold-standard surgical technique for sperm retrieval in patients with NOA, owing to its high sperm retrieval rates and low complication profile. The 2021 American Urological Association (AUA)/American Society for Reproductive Medicine (ASRM) “Diagnosis and Treatment of Infertility in Men” guideline emphasizes the preference for sperm retrieval via m-TESE (Schlegel et al, 2021). The technique enables maximal sperm retrieval (approximately 40–60%) with minimal excisional tissue damage (Kong et al, 2021). Given the substantial inter-individual variability in surgical outcomes, the identification of reliable biomarkers capable of accurately predicting preoperative spermatogenic activity could serve as a valuable clinical guide (Pozzi et al, 2024).Extensive research has focused on the influence of hormonal biomarkers on sperm retrieval outcomes. Owing to their essential roles in spermatogenesis and Sertoli cell maturation, follicle-stimulating hormone (FSH) and inhibin B (INHB) are widely recognized as the most reliable endocrine indicators (Ni, Hao &Yang, 2019). INHB, secreted by Sertoli cells, regulates pituitary FSH release through a negative feedback mechanism. Disruption of seminiferous epithelial integrity correlates with reduced INHB concentrations and elevated FSH levels (Achermann&Esteves, 2023). This reciprocal physiological relationship suggests that the INHB/FSH ratio may provide a more accurate reflection of spermatogenic capacity than either parameter alone.

Objectives
3
State specific objectives, including any prespecified hypotheses
3
The aim of this study is to evaluate the contribution of serum INHB, FSH, and the INHB/FSH ratio in predicting testicular histopathology and to assess their predictive value for sperm retrieval success in NOA patients undergoing m-TESE.

Methods

Study design
4
Present key elements of study design early in the paper
4
retrospective, single-center study
Setting
5
Describe the setting, locations, and relevant dates, including periods of recruitment, exposure, follow-up, and data collection
4
Study was conducted at Elazığ Fethi Sekin City Hospital (Türkiye), between January 2023 and September 2025.
Participants
6
(a) Cohort study—Give the eligibility criteria, and the sources and methods of selection of participants. Describe methods of follow-up
Case-control study—Give the eligibility criteria, and the sources and methods of case ascertainment and control selection. Give the rationale for the choice of cases and controls
Cross-sectional study—Give the eligibility criteria, and the sources and methods of selection of participants


4
Patient Selection
This study included patients diagnosed with NOA who underwent micro-TESE. Demographic, hormonal, and histopathological data were collected retrospectively from the hospital’s electronic medical records.
Inclusion Criteria
Diagnosis of NOA confirmed by at least two semen analyses showing complete absence of spermatozoa after centrifugation.
Aged between 20 to 50 years,
Underwent m-TESE,
Availability of complete preoperative hormonal data (FSH, LH, total testosterone, Inhibin B) measured within two months before surgery.
Availability of histopathological evaluation results after m-TESE.
Exclusion Criteria
Obstructive azoospermia (OA) or ejaculatory duct obstruction diagnosed via clinical, biochemical, or imaging findings.
History of previous testicular surgery, chemotherapy, radiotherapy, or testicular trauma.
Presence of systemic endocrine disorders (e.g., hypogonadotropic hypogonadism, thyroid dysfunction, pituitary adenoma).
Use of exogenous testosterone, anabolic steroids, or hormonal therapy within the past six months.
Incomplete medical records, missing hormonal data, or unavailable histopathology results.
Coexisting genetic abnormalities confirmed through karyotype or molecular analysis.

(b) Cohort study—For matched studies, give matching criteria and number of exposed and unexposed
Case-control study—For matched studies, give matching criteria and the number of controls per case

	6	
All patients (n = 73) who met the inclusion criteria and had complete hormone and histopathological data were included in the analysis during the study period.
Variables
7
Clearly define all outcomes, exposures, predictors, potential confounders, and effect modifiers. Give diagnostic criteria, if applicable
 6
Surgical Technique
The procedure is based on the use of an operating microscope and microsurgical techniques to identify and retrieve seminiferous tubules with a higher likelihood of containing spermatozoa. After adequate sedation was achieved, a midline scrotal incision was made and the testis was delivered through the incision. The tunica vaginalis was opened, and the tunica albuginea was incised under the operating microscope. The seminiferous tubules were examined macroscopically. Tubules that appeared larger and more opaque features suggestive of a higher probability of containing spermatozoa were identified. Small tissue samples were excised using microsurgical scissors with minimal trauma to the surrounding tissue. The excised specimens were simultaneously transferred to the embryology laboratory, where the presence or absence of spermatozoa was confirmed by direct microscopic examination. At the conclusion of the procedure, the tunica albuginea and scrotal layers were closed with sutures. Patients were discharged after their vital signs were stable and an Aldrete score ≥ 9 was achieved, and they were advised to avoid strenuous physical activity for one week.
Histopathological examination
The obtained testicular biopsy specimens were fixed in 10% neutral buffered formalin for at least 6 hours. Following fixation, tissue processing was performed according to the standard histopathological protocol. During dehydration, the specimens were passed through a graded series of ethanol (70%, 96%, and 100%) for a total of approximately 2–3 hours. Subsequently, in the clearing phase, the tissues were treated twice with xylene for a total duration of 40–60 minutes. For paraffin infiltration, the samples were processed in two stages in molten paraffin baths, each lasting approximately 45–60 minutes, and then embedded into paraffin blocks. Sections were cut at a thickness of 3 µm using a rotary microtome, mounted on glass slides via a water bath, and dried at 37°C for at least 24 hours. Hematoxylin and eosin (H&E) staining was employed for routine histological evaluation. Prepared slides were examined under a Leica DM2000 LED light microscope by a single experienced pathologist in a blinded manner. Histopathological diagnoses were established according to the following morphological criteria: Sertoli Cell Only Syndrome (SCOS) was defined by the presence of only Sertoli cells in all seminiferous tubules with a complete absence of germ cells. Early maturation arrest was characterized by cessation of spermatogenesis at the spermatogonium or primary spermatocyte stage, without the presence of more advanced germ cells. Late maturation arrest was defined by germ cell development reaching the spermatid stage, but without mature spermatozoa in the tubules. Hypospermatogenesis was diagnosed when all stages of spermatogenesis were present but with a marked reduction in germ cell numbers and thinning of the germinal epithelium in the seminiferous tubules.
Data sources/ measurement
8*
 For each variable of interest, give sources of data and details of methods of assessment (measurement). Describe comparability of assessment methods if there is more than one group
 6
Histopathological Classification
Based on the results of testicular histopathology, patients were categorized into five groups:
1.Early Maturation Arrest (EMA)
2.Late Maturation Arrest (LMA)
3.Hypospermatogenesis (HS)
4.Normospermatogenesis (NS)
5.Sertoli Cell-Only Syndrome (SCOS)
All evaluations were performed by the same pathologist, blinded to hormonal results, to minimize observer bias.
Hormonal Measurements
Serum hormone levels, including follicle-stimulating hormone (FSH), luteinizing hormone (LH), testosterone, and Inhibin B, were measured using the Beckman Coulter DXI 800 Immunoassay Analyzer based on the chemiluminescence method.
In patients with very low Inhibin B levels, the lowest measurable value (4.6 pg/mL) was used for statistical purposes.
Bias
9
Describe any efforts to address potential sources of bias
 6
All evaluations were performed by the same pathologist, blinded to hormonal results, to minimize observer bias.
Study size
10
Explain how the study size was arrived at
6
All patients (n = 73) who met the inclusion criteria and had complete hormone and histopathological data were included in the analysis during the study period. The study was designed for both descriptive and exploratory purposes.
Continued on next page 
Quantitative variables
11
Explain how quantitative variables were handled in the analyses. If applicable, describe which groupings were chosen and why
6-7
All statistical analyses were performed using SPSS version 25.0 (IBM Corp., Armonk, NY, USA). Descriptive statistics were used to summarize patient characteristics and sperm retrieval rates across pathology groups. Continuous variables were expressed as mean ± standard deviation (SD) or median (interquartile range, IQR), while categorical variables were presented as frequencies and percentages. Comparisons of INHB/FSH ratio among pathology groups were assessed using the Kruskal–Wallis test or one-way ANOVA, depending on data distribution.The diagnostic performance of the INHB/FSH ratio for predicting each pathology group was evaluated using Receiver Operating Characteristic (ROC) curve analysis. The area under the curve (AUC), optimal cut-off values (determined by the Youden index), and corresponding sensitivity and specificity were calculated. To assess the predictive power of the INHB/FSH ratio for sperm retrieval success, binary logistic regression analysis was performed, classifying patients into high- and low-retrieval groups. Additionally, an ordinal logistic regression model was used to evaluate the association between INHB/FSH ratio and the severity of histopathological findings, ordered from SCOS to NS. A p-value of <0.05 was considered statistically significant for all analyses.
Statistical methods
12
(a) Describe all statistical methods, including those used to control for confounding
6-7
All statistical analyses were performed using SPSS version 25.0 (IBM Corp., Armonk, NY, USA). Descriptive statistics were used to summarize patient characteristics and sperm retrieval rates across pathology groups. Continuous variables were expressed as mean ± standard deviation (SD) or median (interquartile range, IQR), while categorical variables were presented as frequencies and percentages. Comparisons of INHB/FSH ratio among pathology groups were assessed using the Kruskal–Wallis test or one-way ANOVA, depending on data distribution.The diagnostic performance of the INHB/FSH ratio for predicting each pathology group was evaluated using Receiver Operating Characteristic (ROC) curve analysis. The area under the curve (AUC), optimal cut-off values (determined by the Youden index), and corresponding sensitivity and specificity were calculated. To assess the predictive power of the INHB/FSH ratio for sperm retrieval success, binary logistic regression analysis was performed, classifying patients into high- and low-retrieval groups. Additionally, an ordinal logistic regression model was used to evaluate the association between INHB/FSH ratio and the severity of histopathological findings, ordered from SCOS to NS. A p-value of <0.05 was considered statistically significant for all analyses.
(b) Describe any methods used to examine subgroups and interactions
6-7

(c) Explain how missing data were addressed
6-7

(d) Cohort study—If applicable, explain how loss to follow-up was addressed
Case-control study—If applicable, explain how matching of cases and controls was addressed
Cross-sectional study—If applicable, describe analytical methods taking account of sampling strategy
6-7

(e) Describe any sensitivity analyses
 6-7

Results
Participants
13*
(a) Report numbers of individuals at each stage of study—eg numbers potentially eligible, examined for eligibility, confirmed eligible, included in the study, completing follow-up, and analysed
 7
The study included a total of 73 patients who underwent m-TESE.
(b) Give reasons for non-participation at each stage


(c) Consider use of a flow diagram
Figure 1.
Uploaded as Figure 1.
Descriptive data
14*
(a) Give characteristics of study participants (eg demographic, clinical, social) and information on exposures and potential confounders
 7 
The study included a total of 73 patients who underwent m-TESE. The mean age of the participants was 32.05 ± 5.71 years (range: 24–47).
(b) Indicate number of participants with missing data for each variable of interest
 

(c) Cohort study—Summarise follow-up time (eg, average and total amount)


Outcome data
15*
Cohort study—Report numbers of outcome events or summary measures over time
 7

Case-control study—Report numbers in each exposure category, or summary measures of exposure


Cross-sectional study—Report numbers of outcome events or summary measures


Main results
16
(a) Give unadjusted estimates and, if applicable, confounder-adjusted estimates and their precision (eg, 95% confidence interval). Make clear which confounders were adjusted for and why they were included
6-7-8

(b) Report category boundaries when continuous variables were categorized
6-7-8

(c) If relevant, consider translating estimates of relative risk into absolute risk for a meaningful time period
6-7-8

Continued on next page 
Other analyses
17
Report other analyses done—eg analyses of subgroups and interactions, and sensitivity analyses
6-7-8
## Results
The study included a total of 73 patients who underwent m-TESE. The mean age of the participants was 32.37 ± 5.67 (range: 22–47) years. The mean serum FSH level was 10.92 ± 10.22 (range: 2.10–58.22)mIU/mL, while the mean Inhibin B concentration was124.56 ± 97.84 (range: 4.6–379.7)pg/mL. The calculated INHB/FSH ratio showed a mean value of 27.14 ± 30.18 (range: 0.12–118.38).Distribution of Pathology Groups and Sperm Retrieval Rates
The distribution of patients across different pathology groups revealed significant variations in sperm retrieval rates (SRR) (Table 1). Among the pathology groups, HSG  exhibited the highest SRR, with 80% of patients (24 out of 30) successfully retrieving sperm. In contrast, the SCOS group had the lowest SRR, with only 10% (2 out of 20) of patients obtaining sperm. EMA demonstrated no sperm retrieval (SR), confirming its poor prognosis for sperm production. The LMA group had a modest SRR of 40%, with 4 out of 10 patients yielding sperm. The NS group had the highest success, with all 5 patients (100%) producing sperm. The overall SRR for all patients in this study was 47.9%, indicating that pathologies such as HSG and NS are associated with higher SR success, while SCOS and EMA present significant challenges (Table 1).ROC Analysis by Pathology Group (INHB/FSH Ratio)
ROC analysis of the INHB/FSH ratio revealed its potential as a reliable biomarker for predicting SR success in various pathology groups (Table 2). The SCOS group demonstrated a high Area Under the Curve (AUC) of 0.89, with a cut-off value of <5.0. This threshold showed strong sensitivity (85%) and specificity (82%) in predicting SCOS, with a highly significant p-value (<0.001). Conversely, the EMA group showed a lower AUC of 0.80, with a cut-off of <15, suggesting that lower INHB/FSH ratios increase the likelihood of EMA, as confirmed by a p-value of 0.004. The LMA group had a moderate predictive value (AUC = 0.77) with a cut-off value of <20, indicating a reasonable but not highly accurate prediction for LMA. The HSG group demonstrated a higher AUC of 0.84, with a cut-off of >10, highlighting the strong predictive capability of higher INHB/FSH ratios for HSG. Finally, NS exhibited the highest AUC (0.91) with a cut-off value of >45, indicating that higher INHB/FSH ratios are strongly associated with NS, further supported by high sensitivity (88%) and specificity (85%).(Table 2, figure 2 )Binary Logistic Regression
Binary logistic regression was performed to classify patients into two groups based on sperm retrieval outcomes:
Low SRR = SCOS, EMA, LMA
High SRR =HSG and NS
The INHB/FSH ratio emerged as a significant predictor of SR, with a p-value of <0.001 (Table 3). For each unit increase in the INHB/FSH ratio, the odds of achieving good spermatogenesis (i.e., hypo-spermatogenesis or normal spermatogenesis) increased by 8-9%. The odds ratio (OR) of 1.089 indicates that higher INHB/FSH ratios are associated with a greater likelihood of SR success. The model’s intercept value of -1.92 suggests a baseline log-odds of SR in the absence of the INHB/FSH ratio, aligning with clinical observations that impaired spermatogenesis is common in certain pathological conditions.(Table 3) Ordinal Regression (Multiclass Pathology Classification)
Ordinal regression was conducted to analyze the relationship between the INHB/FSH ratio and the severity of pathology groups, ranked from SCOS (worst) to NS (best). The INHB/FSH ratio was found to significantly predict pathology severity, with a p-value of <0.001 (Table 4). For each unit increase in the INHB/FSH ratio, there was a significant shift toward less severe pathology groups (e.g., from SCOS to HSG or NS). This finding suggests that a higher INHB/FSH ratio is associated with improved spermatogenesis quality and less severe pathology. The model’s Nagelkerke Pseudo R² value of 0.59 indicates that the model explains a moderate proportion of the variance in the data, while the χ² statistic (41.3, p < 0.001) indicates a strong overall fit of the model. (Table 4)
Both binary and ordinal regression analyses demonstrate that the INHB/FSH ratio is a robust and significant predictor of SR outcomes and pathology severity. The binary logistic regression analysis revealed that a higher INHB/FSH.
Discussion
Key results
18
Summarise key results with reference to study objectives
8-9-10-11

Limitations
19
Discuss limitations of the study, taking into account sources of potential bias or imprecision. Discuss both direction and magnitude of any potential bias
11
Several limitations should be acknowledged. The sample size of certain pathology groups (particularly EMA and NS) was relatively small, which may affect statistical power. Additionally, the cross-sectional design precludes causal inference, and inter-assay variability in hormone measurement may influence reproducibility. Future studies with larger, multicenter cohorts and standardized hormonal assays are warranted to validate these findings and refine the optimal cut-off thresholds.
Interpretation
20
Give a cautious overall interpretation of results considering objectives, limitations, multiplicity of analyses, results from similar studies, and other relevant evidence
11
In conclusion, the serum INHB/FSH ratio represents a reliable, non-invasive biomarker for predicting both histopathological severity and sperm retrieval success in azoospermic men undergoing micro-TESE.
Generalisability
21
Discuss the generalisability (external validity) of the study results
	11
Incorporating this ratio into preoperative evaluation protocols may enhance clinical decision-making, optimize patient selection, and improve overall outcomes in the management of non-obstructive azoospermia.
Other information

Funding
22
Give the source of funding and the role of the funders for the present study and, if applicable, for the original study on which the present article is based
	11	
The authors received no funding for this work.

*Give information separately for cases and controls in case-control studies and, if applicable, for exposed and unexposed groups in cohort and cross-sectional studies.

Note: An Explanation and Elaboration article discusses each checklist item and gives methodological background and published examples of transparent reporting. The STROBE checklist is best used in conjunction with this article (freely available on the Web sites of PLoS Medicine at http://www.plosmedicine.org/, Annals of Internal Medicine at http://www.annals.org/, and Epidemiology at http://www.epidem.com/). Information on the STROBE Initiative is available at www.strobe-statement.org.
